# Effect of increasing cognitive activity participation via calligraphy practice on salient network and central executive network in older adults with subjective cognitive decline: a secondary analysis of a randomized controlled trial

**DOI:** 10.3389/fnagi.2026.1897104

**Published:** 2026-07-24

**Authors:** Jing Li, Yishan Luo, Zhaohua Huo, Lin Shi, Winnie Chiu Wing Chu, Linda Chiu Wa Lam, Allen Ting Chun Lee

**Affiliations:** 1 Department of Psychiatry, Faculty of Medicine, The Chinese University of Hong Kong, Hong Kong, Hong Kong SAR, China; 2 BrainNow Medical Technology Limited, Hong Kong Science and Technology Park, Hong Kong, Hong Kong SAR, China; 3 Department of Imaging and Interventional Radiology, Faculty of Medicine, The Chinese University of Hong Kong, Hong Kong, Hong Kong SAR, China

**Keywords:** calligraphy practice, central executive network, cognitive activities, dementia prevention, salient network, subjective cognitive decline

## Abstract

**Objectives:**

Regular participation in late-life cognitive activities is associated with a reduced risk of dementia. In a prior randomized controlled trial, we demonstrated that increased calligraphy practice improved working memory and strengthened functional connectivity (FC) within the default mode network in older adults with subjective cognitive decline (SCD). This exploratory secondary analysis examined whether increased calligraphy practice also modulates FC in other large-scale brain networks.

**Methods:**

A total of 112 community-dwelling Chinese adults aged 55–75 years with SCD and prior calligraphy experience were randomly assigned to a control or intervention group. The control group maintained their usual calligraphy practice, while the intervention group doubled their weekly practice time for 6 months. Resting-state FC was assessed at baseline and post-intervention using functional magnetic resonance imaging (fMRI). Mixed models for repeated measures were conducted to compare longitudinal FC changes within the salient network and central executive network between groups.

**Results:**

Compared with the control group, doubling calligraphy practice for 6 months was associated with a significantly greater increase in resting-state FC between right rostral prefrontal cortex and right supramarginal gyrus within the salient network (between-group mean difference = 0.12; 95% CI = 0.03–0.22; *p* = 0.01). In pooled analyses of the whole sample, no significant pre-post changes in FCs within SAN were observed. No significant between-group FC changes were observed within the central executive network.

**Conclusion:**

Increased calligraphy practice was associated with localized FC changes within the salient network, extending our prior findings of more distributed FC changes within the default mode network. Together, these findings suggest late-life engagement in cognitive activities may be relevant to large-scale brain network organization in older adults at risk of dementia.

## Highlights

- Spending more time each week practicing calligraphy was linked to positive changes in specific brain connections involved in attention and mental switching.
- These observed changes were not simply due to aging or repeated testing, but were related to increased practice time compared with usual habits.
- The findings suggest that staying cognitively active in later life may help support healthy brain function, and calligraphy may be a simple and safe activity worth further study.

## Introduction

Subjective cognitive decline (SCD) is characterized by self-reported worsening in cognitive function in the absence of objective impairment on standard neuropsychological testing, with symptoms not attributable to other medical or psychiatric conditions (Jessen et al., 2014a). Accumulating evidence suggests that SCD is associated with a higher risk of mild cognitive impairment and dementia and may represent an early prodromal stage of neurodegenerative disorder (Gifford et al., 2014; Jessen et al., 2010, 2014b; Lin et al., 2022; Reisberg et al., 2008, 2010; van Harten et al., 2018). These findings highlight SCD as a potential therapeutic window for interventions aimed at slowing cognitive decline and reducing progression to dementia.

Regular participation in cognitive activities has been proposed as an economical, accessible, and relatively safe non-pharmacological strategy for cognitive maintenance in older adults (Livingston et al., 2020). Such activities typically involve cognitively demanding tasks that engage higher-order processes, including reasoning, problem solving, learning, and executive control (Anatürk et al., 2021; Fujiwara et al., 2009; Parisi et al., 2012; Rodriguez, 2018). As leisure pursuits, they are often intrinsically motivating and enjoyable, supporting sustained participation over time (Cheng et al., 2012; Lam and Cheng, 2013). Cross-sectional studies have consistently reported positive associations between participation in cognitive activities and performance across different cognitive domains (Christensen et al., 1996; Erber and Szuchman, 1996; Luszcz et al., 1997; Van Boxtel et al., 1996). Moreover, large-scale longitudinal studies suggest that higher levels of cognitive activity are associated with an approximately 30%–40% lower risk of developing dementia over 4–5 years, even after adjusting for APOE ε4 status, comorbid physical and psychiatric conditions, and educational attainment (Akbaraly et al., 2009; Lee et al., 2018; Verghese et al., 2003). Analogous to the role of physical exercise in maintaining musculoskeletal and cardiovascular health, regular engagement in cognitive activities may enhance cognitive reserve and slow cognitive decline, although the underlying neurobiological mechanisms are not fully understood.

Calligraphy practice – alongside music, board games, and painting – has long been regarded as a scholarly intellectual activity in the traditional Chinese culture. Many older adults engage in calligraphy regularly as a long-term leisure pursuit. As a cognitively stimulating activity, calligraphy engages multiple high-level processes through intentional and sequential steps. The practice involves preparation of materials requiring planning and attentional control, precise brush handling demanding fine motor coordination, and character production based on prescribed stroke order and structure, engaging working memory, sequencing, and visuospatial processing (Chan et al., 2017; Chen and Kao, 2002). Sustained attention, focus, and controlled breathing are required to maintain balance and fluency during writing, while post-writing review engages evaluation, self-monitoring, and aesthetic judgment (Ishizu and Zeki, 2013; Jacobsen and Beudt, 2017; Salgues et al., 2024). Previous studies have shown that regular calligraphy practice is associated with improvements in several cognitive domains among older adults with mild cognitive impairment, including orientation, attention, working memory, delayed recall, and processing speed (Chan et al., 2018; Kwok et al., 2011). Improvements in orientation and neuropsychiatric symptoms have also been reported in people with Alzheimer’s disease (AD) (Tai et al., 2016).

At the neural systems level, the triple-network model – comprising the default mode network (DMN), salient network (SAN), and central executive network (CEN) – has been widely implicated in cognitive aging and dementia (Joo et al., 2016; Li et al., 2019; Wu et al., 2016; Zhan et al., 2016). Various observational studies indicate that individuals with SCD exhibit altered functional connectivity (FC) within these networks (Brier et al., 2012; Lee et al., 2023; Simic et al., 2014; Uddin, 2015; Xue et al., 2019). In a randomised controlled trial (RCT) examining the effects of increased calligraphy practice on functional brain networks in people with SCD, we examined whether increasing the amount of calligraphy practice beyond participants’ usual level could modify FCs within the DMN. We previously demonstrated that increased calligraphy practice strengthened FCs within the DMN, particularly involving the medial prefrontal cortex (mPFC), hippocampal formation (HF), inferior parietal lobe (IPL), and lateral temporal cortex (LTC) (Lee et al., 2024). Building on these findings, the present secondary analysis aimed to explore whether increased calligraphy practice also modulates FCs within the SAN and CEN.

By extending the investigation beyond the DMN, this study seeks to determine whether increasing engagement in an established cognitive activity in late life is associated with changes in brain network organization among older adults at risk of dementia.

## Materials and methods

### Data sources and study participants

This secondary analysis was based on anonymized data from a two-arm, parallel-group, assessor-blind RCT conducted in Hong Kong from 2020 to 2022 (Lee et al., 2024). The trial was registered with the Chinese Clinical Trial Registry (ChiCTR1900024433) and approved by the Clinical Research Ethics Committee of the University (Ref. No.: 2019.054). Written informed consent was obtained from all subjects before recruitment. The primary trial was conducted and reported according to the Consolidated Standards of Reporting Trials (CONSORT).

A total of 112 community-living Chinese individuals aged 55–75 years were recruited from community centers. All participants met the inclusion criteria for SCD and had regular calligraphy practice, defined as at least 1 h per week during the 6 months prior to enrolment. SCD was defined as (1) cognitive performance within the normal range, operationalized as a score above the locally validated cutoff for mild cognitive impairment in younger-older adults with higher education on the Hong Kong Chinese version of the Montreal Cognitive Assessment (i.e., MoCA > 25) (Wong et al., 2015); and (2) a positive score on the Abbreviated Memory Inventory for Chinese, a locally validated screening tool for SCD (Lui et al., 2006).

Exclusion criteria were: (1) living in a care home; (2) having a history of neurological or psychiatric disorders, including mild cognitive impairment, dementia, stroke, traumatic brain injury, Parkinson’s disease, depression, bipolar affective disorder, psychosis, or substance abuse; (3) taking drugs that affect cognition (e.g., benzodiazepines, medications for dementia); (4) already attending other cognitive intervention programmes or having regular painting (≥1 h per week); (5) significant impairments in communication or writing; (6) no prior calligraphy experience; and (7) having contraindications to magnetic resonance imaging (MRI).

After baseline assessment, participants were randomly assigned in a 1:1 ratio to the intervention or control group using a computer-generated randomization sequence, with allocation concealed until assignment.

### Interventions

Both groups underwent calligraphy practice for 6 months. Participants in the control group maintained their usual practice routine, including frequency, duration, style, and home setting. Participants in the intervention group also practiced under the same conditions as before but were instructed to double their weekly amount of practice.

Adherence to the intervention was monitored by instructing participants to submit a snapshot of their work and to report the duration of each calligraphy handwriting practice session to the research assistant via WhatsApp or email. The research assistant acknowledged receipt of the submissions and documented the reported practice time, but did not provide any feedback on the participants’ work.

### Neuroimaging outcomes

The outcomes of the present secondary analysis were FCs within the SAN and CEN. Resting-state functional magnetic resonance imaging (rs-fMRI) data, together with high-resolution 3D T1-weighted structural MRI data, were acquired using a 3T MRI scanner with an 8-channel SENSE head coil. For each participant, T1-weighted structural images and axial T2-weighted gradient echo-planar functional images were collected. Neuroimaging data were acquired at two time points for both study groups: at baseline (after eligibility screening and informed consent, and prior to intervention initiation) and post-intervention (after completion of the intervention period). The exact scanning dates were recorded in the imaging acquisition logs. rs-fMRI data were pre-processed using CONN 20.b and SPM12, following the pipeline described in the primary study (Lee et al., 2024). The pre-processing steps were conducted in the following order. The first five volumes of each functional time series were discarded to allow for signal equilibration and adaptation to the scanning environment. Head motion and susceptibility distortion correction were performed using SPM realign and unwarp procedure. Functional images were realigned to the first volume of each run using a rigid-body transformation and resampled using B-spline interpolation to correct for motion-related spatial misalignment. Slice-timing correction was then applied to account for temporal differences in slice acquisition. Subsequently, functional images were co-registered to the corresponding high-resolution T1-weighted structural images, and both structural and functional images were spatially normalized to the Montreal Neurological Institute (MNI) space. Spatial smoothing was then performed using an 8 mm full-width at half maximum (FWHM) Gaussian kernel. Head motion was assessed using framewise displacement (FD) derived from realignment parameters. Outlier volumes were identified using CONN’s Artifact Detection Tools (ART), with FD > 0.5 mm and global signal z-score > 3 used as detection thresholds. Participants were excluded if mean FD > 0.5 mm or if more than 20% of volumes were identified as outliers. No participants were excluded in this study due to head motion criteria. Denoising was performed using the CONN standard pipeline, including regression of white matter and cerebrospinal fluid signals, six head motion parameters, identified outlier scans, and linear trends within each run. The denoised BOLD time series were then band-pass filtered (0.01–0.1 Hz).

Based on canonical large-scale network models and established functional connectivity literature, regions of interest (ROIs) for the SAN were pre-specified as the anterior cingulate cortex (ACC), anterior insula (AInsula), rostral prefrontal cortex (RPFC), and supramarginal gyrus (SMG) (Christoff et al., 2003; Corbetta and Shulman, 2002; Menon and Uddin, 2010; Seeley et al., 2007). For the CEN, the pre-specified ROIs included the lateral prefrontal cortex (LPFC) and posterior parietal cortex (PPC) (Menon and Uddin, 2010). ROIs were defined as anatomically-based masks derived from the Automated Anatomical Labeling (AAL) atlas using the WFU PickAtlas toolbox (RRID:SCR_007378). The mean time series for each ROI was extracted by averaging the signal across all voxels within the corresponding anatomical mask.

FCs between ROI pairs were estimated using Pearson correlation coefficients, with higher values indicating stronger connectivity.

### Other variables

Self-reported data obtained in the primary study were used, including age, sex, education level, years of prior calligraphy practice, and physical comorbidities assessed using the Chronic Illness Rating Scale. Structural MRI data were processed using the AccuBrain^®^ system (BrainNow Medical Technology Co., Ltd.) to derive the Alzheimer’s Disease-Resemblance Atrophy Index (AD-RAI) (Cai et al., 2023; Liu et al., 2021). The AD-RAI ranges from 0 to 1, with higher scores indicating greater resemblance to AD-related atrophy patterns.

### Adherence

Adherence rate was calculated as the proportion of actual weekly calligraphy practice time relative to the target weekly practice time.

### Statistical analyses

Statistical analyses were conducted using SPSS Statistics (IBM). Longitudinal changes in FCs between groups were analyzed using mixed models for repeated measures (MMRMs), with time, group, and their interaction as fixed effects, and participants as a random effect to account for within-subject correlations. Analyses followed the intention-to-treat principle. Missing post-intervention data were imputed using the baseline observation carried forward method, assuming no change from baseline. Sensitivity analyses were conducted among study completers. Within-subject FC changes from baseline to post-intervention were assessed using paired *t*-tests. Exploratory analyses evaluated potential moderators of FC changes, including age, sex, years of education, years of prior calligraphy practice, and baseline AD-RAI scores.

## Results

### Baseline characteristics

Among the 112 participants who provided written informed consent and completed baseline assessment, five (4.5%) dropped out during the intervention, and 96 (85.7%) completed the post-intervention fMRI scan. The mean age of participants was 66.3 years (SD = 4.3); 83 (74.1%) were female, with a mean educational attainment of 14.8 years (SD = 3.6). Participants had engaged in calligraphy practice for a mean of 9.7 years and reported an average practice duration of 3.1 h per week in the 6 months prior to enrolment. The baseline mean MoCA score was 28.5 (SD = 1.2), and the mean AD-RAI was 0.12 (SD = 0.16). The CONSORT flow diagram and detailed baseline characteristics – including age, sex, education, prior calligraphy practice, MoCA score, physical comorbidities and severity of brain atrophy – have been described in the primary trial publication.

### Neuroimaging outcomes

**FIGURE 1:**
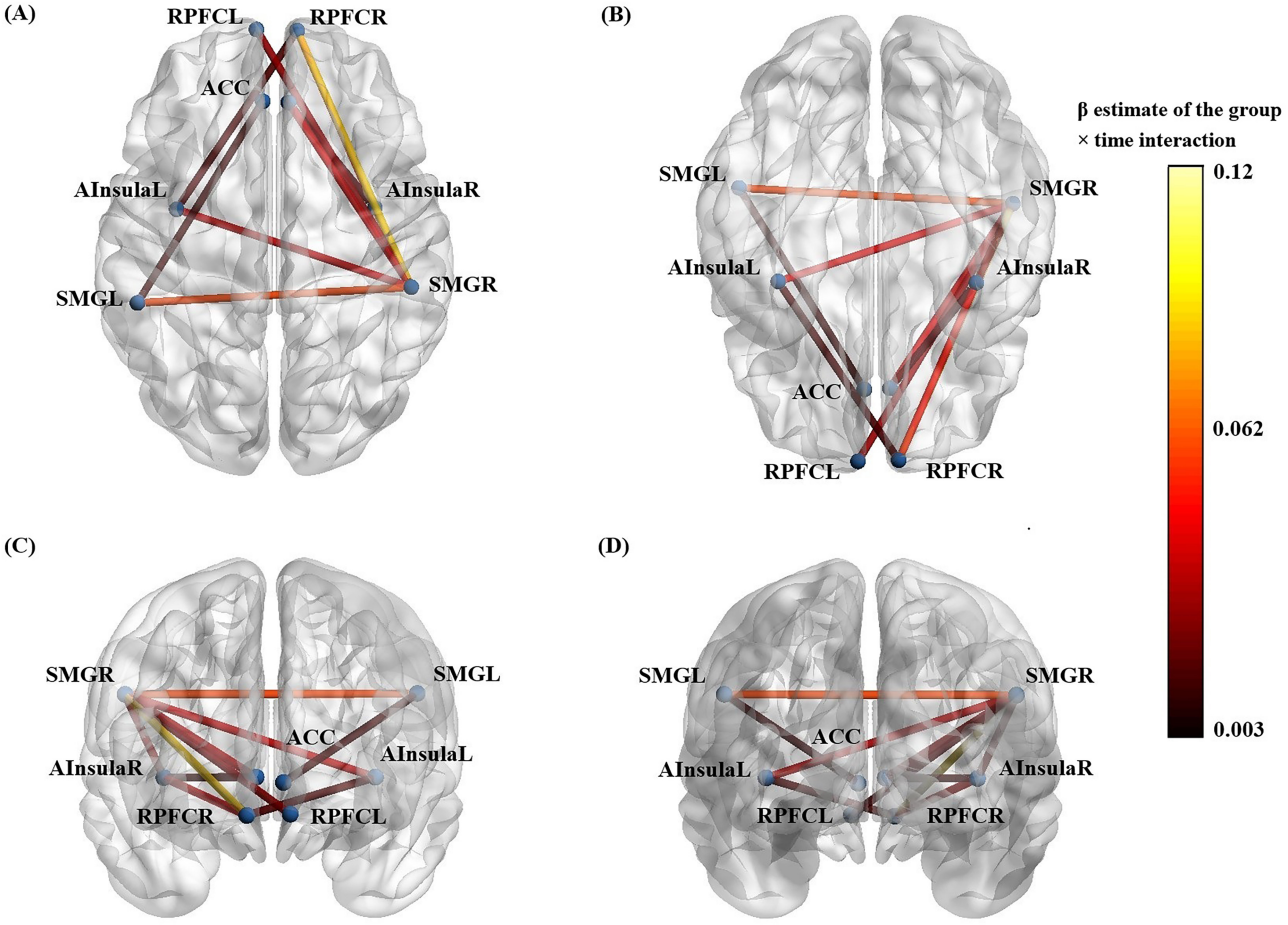
Increased resting-state FCs within the salient network in the intervention group compared with the control group following 6-months calligraphy intervention. **(A)** Axial view; **(B)** inferior view; **(C)** coronal view, **(D)** posterior view. ACC, anterior cingulate cortex; AInsula, anterior insula; RPFC, rostral prefrontal cortex; SMG, supramarginal gyrus; nodes that end with L, left side; nodes that end with R, right side.

Within the SAN, a significant between-group difference was observed in longitudinal FC changes between the right RPFC and right SMG. Compared with the control group, the intervention group showed a greater increase in FC over 6 months (intervention = 0.08 *versus* control = −0.04; group difference = 0.12; 95% CI = 0.03–0.22; *p* = 0.01). While the intervention group also demonstrated greater increases in FC than the control group for several other ROI pairs – including ACC and right AInsula, ACC and right SMG, left AInsula and right SMG, right AInsula and left RPFC, right Ainsula and right RPFC, right AInsula and right SMG, left RPFC and right SMG, and left SMG and right SMG – none of these comparisons showed statistically significant between-group differences in FC change (Figure 1 and Table 1).

**TABLE 1:** Comparison of 6-months changes in functional connectivity of salience network using intention-to-treat mixed models for repeated measures (MMRMs).

Functional connectivity	Intervention group			Control group			Intervention vs. control	
	Mean (SD)		Mean change (95% CI)	Mean (SD)		Mean change (95% CI)	Mean difference (95% CI)	*P*-value
	Baseline	6 months		Baseline	6 months			
ACC_AInsulaL	0.32 (0.23)	0.27 (0.28)	−0.05 (−0.11 to 0.02)	0.31 (0.25)	0.30 (0.24)	−0.01 (−0.08 to 0.06)	−0.03 (−0.13 to 0.06)	0.47
ACC_AInsulaR	0.21 (0.23)	0.23 (0.20)	0.02 (−0.05 to 0.09)	0.25 (0.22)	0.25 (0.22)	0.01 (−0.05 to 0.07)	0.01 (−0.08 to 0.10)	0.79
ACC_RPFCL	0.20 (0.21)	0.22 (0.24)	0.02 (−0.05 to 0.09)	0.18 (0.21)	0.22 (0.24)	0.04 (−0.02 to 0.10)	−0.02 (−0.11 to 0.07)	0.62
ACC_RPFCR	0.17 (0.21)	0.17 (0.25)	0.005 (−0.06 to 0.07)	0.13 (0.24)	0.17 (0.20)	0.04 (−0.02 to 0.11)	−0.04 (−0.13 to 0.05)	0.41
ACC_SMGL	0.20 (0.21)	0.19 (0.26)	−0.004 (−0.07 to 0.06)	0.17 (0.26)	0.17 (0.22)	−0.01 (−0.08 to 0.06)	0.003 (−0.09 to 0.10)	0.95
ACC_SMGR	0.20 (0.19)	0.21 (0.20)	0.01 (−0.06 to 0.08)	0.22 (0.19)	0.19 (0.25)	−0.04 (−0.10 to 0.03)	0.05 (−0.05 to 0.14)	0.33
AInsulaL_AInsulaR	0.41 (0.24)	0.39 (0.23)	−0.01 (−0.08 to 0.05)	0.41 (0.20)	0.42 (0.21)	0.01 (−0.05 to 0.07)	−0.02 (−0.11 to 0.07)	0.64
AInsulaL_RPFCL	0.41 (0.21)	0.42 (0.24)	0.01 (−0.05 to 0.07)	0.41 (0.23)	0.46 (0.18)	0.06 (−0.01 to 0.12)	−0.05 (−0.14 to 0.04)	0.29
AInsulaL_RPFCR	0.47 (0.19)	0.45 (0.23)	−0.02 (−0.09 to 0.04)	0.42 (0.22)	0.43 (0.20)	0.01 (−0.05 to 0.07)	−0.03 (−0.12 to 0.06)	0.49
AInsulaL_SMGL	0.46 (0.16)	0.46 (0.21)	0.01 (−0.05 to 0.06)	0.40 (0.22)	0.43 (0.21)	0.03 (−0.03 to 0.09)	−0.02 (−0.10 to 0.06)	0.55
AInsulaL_SMGR	0.23 (0.25)	0.28 (0.20)	0.05 (−0.02 to 0.12)	0.25 (0.22)	0.25 (0.22)	−0.003 (−0.07 to 0.06)	0.05 (−0.04 to 0.15)	0.26
AInsulaR_RPFCL	0.45 (0.26)	0.50 (0.23)	0.05 (−0.01 to 0.10)	0.53 (0.22)	0.51 (0.20)	−0.02 (−0.06 to 0.03)	0.06 (−0.01 to 0.13)	0.07
AInsulaR_RPFCR	0.36 (0.21)	0.36 (0.23)	0.005 (−0.06 to 0.07)	0.33 (0.24)	0.32 (0.23)	−0.01 (−0.07 to 0.05)	0.01 (−0.08 to 0.10)	0.75
AInsulaR_SMGL	0.21 (0.22)	0.22 (0.21)	0.02 (−0.05 to 0.09)	0.19 (0.23)	0.21 (0.24)	0.03 (−0.03 to 0.09)	−0.01 (−0.10 to 0.09)	0.84
AInsulaR_SMGR	0.40 (0.20)	0.41 (0.19)	0.01 (−0.05 to 0.07)	0.43 (0.20)	0.42 (0.18)	−0.01 (−0.07 to 0.04)	0.02 (−0.06 to 0.10)	0.61
RPFCL_RPFCR	0.20 (0.30)	0.20 (0.30)	−0.001 (−0.08 to 0.08)	0.21 (0.27)	0.23 (0.24)	0.02 (−0.05 to 0.09)	−0.02 (−0.13 to 0.09)	0.70
RPFCL_SMGL	0.44 (0.23)	0.44 (0.20)	−0.002 (−0.06 to 0.06)	0.41 (0.23)	0.45 (0.21)	0.04 (−0.03 to 0.11)	−0.04 (−0.13 to 0.05)	0.38
RPFCL_SMGR	0.32 (0.29)	0.37 (0.23)	0.06 (−0.01 to 0.13)	0.35 (0.27)	0.37 (0.21)	0.02 (−0.05 to 0.10)	0.03 (−0.07 to 0.14)	0.52
RPFCR_SMGL	0.51 (0.20)	0.52 (0.19)	0.01 (−0.04 to 0.06)	0.50 (0.19)	0.55 (0.21)	0.05 (−0.02 to 0.12)	−0.04 (−0.13 to 0.05)	0.40
RPFCR_SMGR	0.35 (0.27)	0.43 (0.23)	0.08 (0.001 to 0.17)	0.41 (0.22)	0.37 (0.23)	−0.04 (−0.09 to 0.01)	0.12 (0.03 to 0.22)	**0.01**
SMGL_SMGR	0.31 (0.28)	0.37 (0.21)	0.07 (0.002 to 0.13)	0.30 (0.26)	0.28 (0.22)	−0.01 (−0.09 to 0.06)	0.08 (−0.02 to 0.17)	0.10

ACC, anterior cingulate cortex; AInsula, anterior insula; RPFC, rostral prefrontal cortex; SMG, supramarginal gyrus; nodes that end with L, left side; nodes that end with R, right side. Value shown in bold denotes statistical significance at the 0.05 level (*p* < 0.05).

No significant between-group FC changes were observed within the CEN (Table 2).

**TABLE 2:** Comparison of 6-months changes in functional connectivity of central executive network using intention-to-treat mixed models for repeated measures (MMRMs).

Functional connectivity	Intervention group			Control group			Intervention vs. control	
	Mean (SD)		Mean change (95% CI)	Mean (SD)		Mean change (95% CI)	Mean difference (95% CI)	*P*-value
	Baseline	6 months		Baseline	6 months			
LPFCL_LPFCR	0.14 (0.24)	0.10 (0.25)	−0.03 (−0.12 to 0.05)	0.11 (0.20)	0.11 (0.23)	−0.001 (−0.08 to 0.08)	−0.03 (−0.15 to 0.08)	0.56
LPFCL_PPCL	0.12 (0.22)	0.15 (0.24)	0.03 (−0.05 to 0.11)	0.09 (0.20)	0.12 (0.20)	0.03 (−0.03 to 0.10)	−0.001 (−0.10 to 0.10)	0.99
LPFCL_PPCR	0.14 (0.25)	0.10 (0.24)	−0.04 (−0.12 to 0.04)	0.10 (0.24)	0.12 (0.24)	0.02 (−0.06 to 0.09)	−0.06 (−0.16 to 0.05)	0.31
LPFCR_PPCR	0.04 (0.32)	0.08 (0.31)	0.04 (−0.06 to 0.14)	0.05 (0.28)	0.04 (0.32)	−0.01 (−0.09 to 0.07)	0.05 (−0.08 to 0.17)	0.44
PPCL_PPCR	0.05 (0.34)	0.13 (0.31)	0.08 (−0.02 to 0.18)	0.04 (0.34)	0.15 (0.34)	0.11 (−0.01 to 0.23)	−0.02 (−0.18 to 0.13)	0.75
PPCL_LPFCR	0.50 (0.26)	0.47 (0.23)	−0.03 (−0.11 to 0.05)	0.48 (0.21)	0.53 (0.20)	0.05 (−0.03 to 0.13)	−0.08 (−0.19 to 0.04)	0.18

LPFC, lateral prefrontal cortex; PPC, posterior parietal cortex; nodes that end with L, left side; nodes that end with R, right side.

### Sensitivity and exploratory analyses

Sensitivity analyses restricted to participants who completed the intervention yielded results consistent with the intention-to-treat analyses (Supplementary eTables 1, 2 in online Supplementary Material). In the SAN, a significant between-group difference was observed in longitudinal FC changes between the right RPFC and right SMG. Compared with the control group, the intervention group showed a greater increase in FC over 6 months (intervention = 0.10 versus control = −0.05; group difference = 0.15; 95% CI = 0.04–0.26; *p* = 0.01). In pooled analyses of the whole sample, no significant pre-post changes in SAN FCs were observed (Supplementary eTable 3).

Exploratory subgroup analyses suggested heterogeneity in SAN FC changes associated with increased calligraphy practice (Supplementary eTables 4–8). Subgroups were defined *a priori* based on baseline distributions of demographic and clinical variables.

The significant FC increase between the right RPFC and right SMG within the SAN was observed in older participants (aged 66–75 years; intervention = 0.14 *versus* control = −0.05; group difference = 0.19; 95% CI = 0.03–0.35; *p* = 0.01) but not in younger participants (aged 55–65 years; Supplementary eTable 4), and in females (intervention = 0.09 *versus* control = −0.04; group difference = 0.13; 95% CI = 0.02–0.25; *p* = 0.02) but not in males (Supplementary eTable 5).

When stratified by educational attainment, significant FC changes were observed in distinct connections across both lower- and higher-education subgroups (Supplementary eTable 6). In the lower-education subgroup, a significant FC increase was observed between the ACC and right SMG (intervention = 0.01 *versus* control = −0.15; group difference = 0.16; 95% CI = 0.01–0.30; *p* = 0.03). In the higher-education subgroup, significant FC increases were observed between the right RPFC and right SMG (intervention = 0.13 *versus* control = −0.05; group difference = 0.18; 95% CI = 0.05–0.31; *p* = 0.01), and between the left SMG and right SMG (intervention = 0.11 *versus* control = −0.02; group difference = 0.13; 95% CI = 0.001–0.26; *p* = 0.049).

No significant FC changes were observed when stratified by years of prior calligraphy practice (Supplementary eTable 7).

Interestingly, significant FC increases within the SAN were identified among participants with greater baseline brain atrophy (Supplementary eTable 8). Participants were stratified into two subgroups according to baseline AD-RAI values. In the greater-atrophy subgroup, significant FC increases were observed between the right RPFC and right SMG (intervention = 0.11 *versus* control = −0.05; group difference = 0.16; 95% CI = 0.01–0.31; *p* = 0.03), and between the left SMG and right SMG (intervention = 0.07 *versus* control = −0.07; group difference = 0.14; 95% CI = 0.01–0.27; *p* = 0.04).

### Adherence

Median adherence rates were 100.0% (IQR = 79.3%–109.6%) in the intervention group and 107.0% (IQR = 94.8%–126.8%) in the control group. The target weekly practice time was 5.5 (SD = 4.0) hours in the intervention group and 3.4 (SD = 2.3) hours in the control group, compared with actual practice times of 5.7 (SD = 3.4) and 3.7 (SD = 2.7) hours per week, respectively. Actual weekly practice time was significantly greater in the intervention group than in the control group (*p* = 0.002).

## Discussion

In this secondary analysis of a randomized controlled trial, increased engagement in calligraphy practice was associated with selective changes in large-scale brain network connectivity in older adults with SCD. Extending our primary findings of enhanced FCs within the DMN, we observed a significant but localized between-group increase within the SAN, alongside no detectable changes within the CEN. Pooled analyses of the whole sample showed no overall pre-post changes in SAN connectivity, indicating that the observed effects were driven by differential trajectories between the intervention and control groups rather than a general time effect. Taken together, these findings suggest that increasing engagement in a cognitively stimulating activity in late life may be associated with network-specific functional modulation across brain systems.

### Differential effects on large-scale brain networks

The SAN functions as a key hub for detecting salient internal or external signals and facilitating dynamic switching between the DMN and CEN (Menon and Uddin, 2010; Seeley et al., 2007; Sridharan et al., 2008). The relatively limited SAN modulation observed in the present analysis – restricted to FC between the rostral prefrontal cortex and supramarginal gyrus – may reflect intermittent demands for attentional monitoring and control during calligraphy practice, such as evaluating stroke precision and adjusting motor output, requiring transient shifts between internally focused processing and task-directed control. The absence of a global SAN time effect in pooled analyses further supports the interpretation that these changes represent intervention-specific modulation of network coordination rather than general changes over time.

The lack of significant changes within the CEN may reflect the high level of skill expertise and the automatized nature of task performance in the present cohort. Calligraphy practice in this cohort involved highly practiced motor sequences, with participants averaging nearly a decade of prior experience. Unlike novel or cognitively demanding problem-solving activities that robustly engaging the CEN (Gerlach et al., 2011), such familiar practices impose limited sustained executive control demands. This interpretation aligns with prior evidence that as skills become automatized, reliance on CEN diminishes in favor of more efficient, internally guided processing (Floyer-Lea and Matthews, 2004; Poldrack et al., 2005).

Within the triple-network framework, these findings can be integrated into a broader systems-level interpretation. Overall, a gradient of FC changes was observed across large-scale networks, with the most prominent effects in the DMN, more circumscribed effects in the SAN, and no significant changes in the CEN. The DMN supports internally oriented cognitive processes such as autobiographical memory retrieval (Ino et al., 2011; Philippi et al., 2015; Yang et al., 2013), visuospatial imagery (Hassabis and Maguire, 2007), motor sequence learning (Eryurek et al., 2022; Hendrikse et al., 2025; Jacobacci et al., 2020), and self-referential processing (Davey et al., 2016; Vatansever et al., 2018), all of which are integral to calligraphy practice. Producing characters requires sustained internal visualization of stroke structure, retrieval of learned motor schemas, sequential coordination of movements, and continuous self-monitoring of accuracy and aesthetics, consistent with the distributed DMN connectivity enhancements observed in the primary trial.

### Implications for cognitive activity and cognitive reserve

Viewed within the cognitive reserve framework (Stern et al., 2019), these findings support the notion that sustained engagement in cognitively stimulating activities may be relevant to the organization and adaptability of large-scale brain networks implicated in early, preclinical stage of neurodegeneration. While previous studies have reported cognitive and psychological benefits of calligraphy practice across the lifespan (Chen et al., 2017, 2019, 2024; Kao et al., 2014; Wang and Tang, 2024, 2025; Wang Z. et al., 2023; Wang Y. et al., 2023), many did not account for prior experience or disentangle intervention-specific effects from general motor or visual engagement. The present trial addresses several of these limitations through experimental manipulation of practice volume, exclusion of other structured cognitive activities, explicit assessment of prior exposure, and longitudinal analyses, thereby strengthening causal inference beyond observational designs.

Notably, the findings suggest that even among individuals with long-term engagement in calligraphy practice, a modest increase in practice volume over 6 months may be associated with detectable neural changes. This observation is consistent with a dose-dependent model of cognitive activity and suggests that habitual engagement does not preclude further network-level plasticity in later life.

### Exploratory subgroup heterogeneity

Exploratory subgroup analyses suggested variations in SAN connectivity changes across participant characteristics. Significant between-group effects were observed among older participants, women, and individuals with greater baseline brain atrophy, but not among younger participants, men, or those with lesser atrophy. Educational attainment appeared to influence the specific SAN connections involved, whereas years of prior calligraphy experience did not. These patterns may reflect differences in baseline network vulnerability, variability, or measurement sensitivity rather than differential intervention efficacy. In addition, unequal subgroup sizes and limited statistical power – particularly given the higher proportion of female participants – may have contributed to the observed heterogeneity. As these analyses were exploratory and not powered for formal interaction testing, the findings should be regarded as hypothesis-generating and warrant confirmation in larger, adequately powered studies.

### Study strengths and limitations

This study benefits from a well-characterized RCT design, with rigorous screening for SCD, careful exclusion of mimicking conditions, and balanced baseline characteristics between groups. In addition, the use of pre- and post-intervention FC outcomes within a theoretically grounded triple-network framework provides objective, mechanistically informative markers of brain network organization beyond behavioral measures alone.

Nevertheless, this study has several limitations. First, the sample consisted of well-educated, community-living Chinese older adults with longstanding calligraphy experience, which may limit generalizability to calligraphy-naïve individuals or other cultural contexts. Second, the analyses focused on resting state FCs; future studies incorporating dynamic connectivity approaches may better capture time-varying network interactions relevant to early cognitive decline. Third, as a secondary analysis, this study was not prospectively powered to detect network-specific effects within the SAN or CEN. Fourth, although a neuroimaging-based AD-RAI was included, the absence of molecular biomarkers limits direct inference regarding AD-specific neuropathological processes. Finally, adherence was monitored using self-reported practice duration supplemented by submission of calligraphy samples. However, the actual duration of practice could not be independently verified and may be susceptible to reporting bias.

## Conclusion

Increased calligraphy practice was associated with localized FC changes within the SAN, complementing previously reported, more widespread modulation of the DMN. Collectively, these findings suggest that late-life engagement in cognitive activities may be relevant to the organization of large-scale brain networks in older adults at risk of dementia. As a low-cost, safe, and highly acceptable activity, calligraphy practice represents a feasible lifestyle-based approach that warrants further investigation in dementia risk-reduction research.

## Data Availability

The datasets presented in this article are not readily available due to ethical and institutional restrictions. Requests to access the datasets should be directed to AL, allenlee@cuhk.edu.hk.
