# Supplementary material for: Effect of increasing cognitive activity participation via calligraphy practice on salient network and central executive network in older adults with subjective cognitive decline: a secondary analysis of a randomized controlled trial
Source: Front Aging Neurosci. 2026 Jul 24;18:1897104. doi: 10.3389/fnagi.2026.1897104 (PMC13447491; doi:10.3389/fnagi.2026.1897104)
Supplement: Supplementary file 1 [file Data_Sheet_1.docx]

**Online-Only Supplementary Materials**

[**eTable 1. Comparison of 6-month changes in functional connectivity of salience network using per-protocol mixed models for repeated measures (MMRMs).**	2](#_Toc235316378)

[**eTable 2.Comparison of 6-month changes in functional connectivity of central executive network using per-protocol mixed models for repeated measures (MMRMs).**	4](#_Toc235316379)

[**eTable 3. Comparison of functional connectivity of salience network before and after intervention using paired t test.**	5](#_Toc235316380)

[**eTable 4. Effect of doubling calligraphy practice on functional connectivity changes of salience network in younger and older age groups.**	7](#_Toc235316381)

[**eTable 5. Effect of doubling calligraphy practice on functional connectivity changes of salience network in female and male groups.**	9](#_Toc235316382)

[**eTable 6. Effect of doubling calligraphy practice on functional connectivity changes of salience network in groups with fewer and more years of education.**	11](#_Toc235316383)

[**eTable 7. Effect of doubling calligraphy practice on functional connectivity changes of salience network in groups with fewer and more of prior practice.**	13](#_Toc235316384)

[**eTable 8. Effect of doubling calligraphy practice on functional connectivity changes of salience network in groups of lesser and greater brain atrophy.**	15](#_Toc235316385)

**eTable 1. Comparison of 6-month changes in functional connectivity of salience network using per-protocol mixed models for repeated measures (MMRMs).**

| **Functional Connectivity** | **Intervention group (n = 48)** | | | **Control group (n = 48)** | | | **Intervention vs Control** | |
| --- | --- | --- | --- | --- | --- | --- | --- | --- |
|  | **Mean (SD)** | | **Mean change**  **(95% CI)** | **Mean (SD)** | | **Mean change**  **(95% CI)** | **Mean difference**  **(95% CI)** | **p-value** |
|  | **Baseline** | **6 months** |  | **Baseline** | **6 months** |  |  |  |
| ACC_AInsulaL | 0.30 (0.23) | 0.25 (0.29) | -0.05  (-0.14 – 0.03) | 0.31 (0.26) | 0.30 (0.25) | -0.01  (-0.10 – 0.07) | -0.04  (-0.15 – 0.07) | 0.47 |
| ACC_AInsulaR | 0.20 (0.23) | 0.22 (0.19) | 0.02  (-0.06 – 0.11) | 0.25 (0.21) | 0.26 (0.21) | 0.01  (-0.07 – 0.08) | 0.01  (-0.10 – 0.13) | 0.79 |
| ACC_RPFCL | 0.19 (0.22) | 0.21 (0.25) | 0.02  (-0.06 – 0.10) | 0.19 (0.21) | 0.24 (0.24) | 0.05  (-0.02 – 0.12) | -0.03  (-0.14 – 0.08) | 0.61 |
| ACC_RPFCR | 0.14 (0.20) | 0.14 (0.25) | 0.01  (-0.07 – 0.08) | 0.14 (0.26) | 0.19 (0.21) | 0.05  (-0.03 – 0.13) | -0.05  (-0.15 – 0.06) | 0.40 |
| ACC_SMGL | 0.18 (0.20) | 0.17 (0.26) | -0.005  (-0.08 – 0.07) | 0.18 (0.28) | 0.17 (0.23) | -0.01  (-0.09 – 0.08) | 0.004  (-0.11 – 0.12) | 0.95 |
| ACC_SMGR | 0.18 (0.20) | 0.20 (0.21) | 0.01  (-0.07 – 0.10) | 0.23 (0.18) | 0.19 (0.25) | -0.05  (-0.13 – 0.04) | 0.06  (-0.06 – 0.17) | 0.32 |
| AInsulaL_AInsulaR | 0.41 (0.23) | 0.40 (0.21) | -0.02  (-0.09 – 0.06) | 0.40 (0.20) | 0.40 (0.22) | 0.01  (-0.07 – 0.08) | -0.02  (-0.13 – 0.08) | 0.64 |
| AInsulaL_RPFCL | 0.43 (0.19) | 0.44 (0.22) | 0.01  (-0.06 – 0.08) | 0.39 (0.24) | 0.46 (0.18) | 0.07  (-0.01 – 0.15) | -0.06  (-0.17 – 0.05) | 0.28 |
| AInsulaL_RPFCR | 0.44 (0.18) | 0.41 (0.23) | -0.03  (-0.10 – 0.05) | 0.41 (0.23) | 0.42 (0.21) | 0.01  (-0.06 – 0.09) | -0.04  (-0.14 – 0.07) | 0.49 |
| AInsulaL_SMGL | 0.44 (0.16) | 0.44 (0.21) | 0.01  (-0.06 – 0.07) | 0.39 (0.23) | 0.43 (0.22) | 0.04  (-0.04 – 0.11) | -0.03  (-0.12 – 0.07) | 0.55 |
| AInsulaL_SMGR | 0.22 (0.27) | 0.28 (0.21) | 0.06  (-0.02 – 0.14) | 0.25 (0.22) | 0.25 (0.21) | -0.003  (-0.08 – 0.08) | 0.06  (-0.05 – 0.18) | 0.27 |
| AInsulaR_RPFCL | 0.47 (0.22) | 0.53 (0.18) | 0.05  (-0.01 – 0.12) | 0.52 (0.23) | 0.50 (0.21) | -0.02  (-0.08 – 0.04) | 0.08  (-0.01 – 0.16) | 0.07 |
| AInsulaR_RPFCR | 0.36 (0.20) | 0.36 (0.22) | 0.01  (-0.08 – 0.09) | 0.34 (0.24) | 0.33 (0.24) | -0.01  (-0.08 – 0.06) | 0.02  (-0.09 – 0.13) | 0.75 |
| AInsulaR_SMGL | 0.20 (0.23) | 0.22 (0.21) | 0.02  (-0.06 – 0.10) | 0.19 (0.23) | 0.22 (0.24) | 0.03  (-0.04 – 0.10) | -0.01  (-0.12 – 0.10) | 0.83 |
| AInsulaR_SMGR | 0.42 (0.19) | 0.43 (0.18) | 0.01  (-0.06 – 0.08) | 0.44 (0.20) | 0.43 (0.17) | -0.02  (-0.08 – 0.05) | 0.03  (-0.07 – 0.12) | 0.61 |
| RPFCL_RPFCR | 0.22 (0.26) | 0.22 (0.26) | -0.001  (-0.10 – 0.10) | 0.19 (0.27) | 0.21 (0.23) | 0.02  (-0.06 – 0.11) | -0.02  (-0.15 – 0.10) | 0.70 |
| RPFCL_SMGL | 0.47 (0.21) | 0.47 (0.19) | -0.003  (-0.08 – 0.07) | 0.40 (0.23) | 0.45 (0.21) | 0.05  (-0.04 – 0.13) | -0.05  (-0.16 – 0.06) | 0.37 |
| RPFCL_SMGR | 0.35 (0.28) | 0.42 (0.18) | 0.07  (-0.02 – 0.16) | 0.34 (0.28) | 0.37 (0.21) | 0.03  (-0.06 – 0.12) | 0.04  (-0.09 – 0.16) | 0.53 |
| RPFCR_SMGL | 0.51 (0.20) | 0.52 (0.19) | 0.01  (-0.05 – 0.07) | 0.48 (0.20) | 0.54 (0.21) | 0.06  (-0.03 – 0.14) | -0.05  (-0.15 – 0.06) | 0.39 |
| RPFCR_SMGR | 0.32 (0.27) | 0.42 (0.23) | 0.10  (0.001 – 0.20) | 0.41 (0.22) | 0.36 (0.23) | -0.05  (-0.11 – 0.01) | 0.15  (0.04 – 0.26) | **0.01** |
| SMGL_SMGR | 0.30 (0.29) | 0.38 (0.20) | 0.08  (0.002 – 0.15) | 0.31 (0.26) | 0.30 (0.22) | -0.02  (-0.10 – 0.07) | 0.09  (-0.02 – 0.21) | 0.10 |

Abbreviations: ACC – Anterior cingulate cortex; AInsula – Anterior insula; RPFC – Rostral prefrontal cortex; SMG – Supramarginal gyrus; nodes that end with L – left side; nodes that end with R – right side.

**eTable 2.Comparison of 6-month changes in functional connectivity of central executive network using per-protocol mixed models for repeated measures (MMRMs).**

| **Functional Connectivity** | **Intervention group (n = 48)** | | | **Control group (n = 48)** | | | **Intervention vs Control** | |
| --- | --- | --- | --- | --- | --- | --- | --- | --- |
|  | **Mean (SD)** | | **Mean change**  **(95% CI)** | **Mean (SD)** | | **Mean change**  **(95% CI)** | **Mean difference**  **(95% CI)** | **p-value** |
|  | **Baseline** | **6 months** |  | **Baseline** | **6 months** |  |  |  |
| LPFCL_LPFCR | 0.12 (0.24) | 0.08 (0.25) | -0.04  (-0.14 – 0.06) | 0.10 (0.20) | 0.10 (0.24) | -0.001  (-0.09 – 0.09) | -0.04  (-0.18 – 0.09) | 0.55 |
| LPFCL_PPCL | 0.10 (0.22) | 0.13 (0.24) | 0.04  (-0.06 – 0.13) | 0.09 (0.20) | 0.13 (0.21) | 0.04  (-0.04 – 0.12) | -0.001  (-0.12 – 0.12) | 0.998 |
| LPFCL_PPCR | 0.13 (0.25) | 0.09 (0.24) | -0.05  (-0.14 – 0.05) | 0.08 (0.26) | 0.11 (0.25) | 0.02  (-0.07 – 0.11) | -0.07  (-0.20 – 0.06) | 0.31 |
| LPFCR_PPCR | 0.04 (0.31) | 0.09 (0.30) | 0.05  (-0.07 – 0.16) | 0.05 (0.27) | 0.04 (0.32) | -0.01  (-0.11 – 0.08) | 0.06  (-0.09 – 0.21) | 0.44 |
| PPCL_PPCR | 0.04 (0.34) | 0.14 (0.30) | 0.10  (-0.02 – 0.22) | 0.02 (0.34) | 0.15 (0.34) | 0.13  (-0.01 – 0.27) | -0.03  (-0.22 – 0.15) | 0.73 |
| PPCL_LPFCR | 0.51 (0.27) | 0.47 (0.24) | -0.04  (-0.13 – 0.06) | 0.45 (0.23) | 0.51 (0.22) | 0.06  (-0.03 – 0.15) | -0.10  (-0.23 – 0.04) | 0.17 |

Abbreviations: LPFC – Lateral prefrontal cortex; PPC – Posterior parietal cortex; nodes that end with L – left side; nodes that end with R – right side.

**eTable 3. Comparison of functional connectivity of salience network before and after intervention using paired t test.**

| **Functional Connectivity** | **Whole sample** | | | |
| --- | --- | --- | --- | --- |
|  | **Mean (SD)** | | **Mean change (95% CI)** | **p-value** |
|  | **Baseline** | **6 months** |  |  |
| ACC_AInsulaL | 0.31 (0.24) | 0.29 (0.26) | -0.03 (-0.08 – 0.02) | 0.24 |
| ACC_AInsulaR | 0.23 (0.23) | 0.24 (0.21) | 0.01 (-0.03 – 0.06) | 0.59 |
| ACC_RPFCL | 0.19 (0.21) | 0.22 (0.24) | 0.03 (-0.02 – 0.07) | 0.22 |
| ACC_RPFCR | 0.15 (0.23) | 0.17 (0.22) | 0.02 (-0.02 – 0.07) | 0.30 |
| ACC_SMGL | 0.19 (0.24) | 0.18 (0.24) | -0.01 (-0.05 – 0.04) | 0.82 |
| ACC_SMGR | 0.21 (0.19) | 0.20 (0.22) | -0.01 (-0.06 – 0.04) | 0.59 |
| AInsulaL_AInsulaR | 0.41 (0.22) | 0.41 (0.22) | -0.004 (-0.05 – 0.04) | 0.84 |
| AInsulaL_RPFCL | 0.41 (0.22) | 0.44 (0.21) | 0.03 (-0.01 – 0.08) | 0.17 |
| AInsulaL_RPFCR | 0.45 (0.20) | 0.44 (0.22) | -0.01 (-0.05 – 0.04) | 0.78 |
| AInsulaL_SMGL | 0.43 (0.20) | 0.44 (0.21) | 0.02 (-0.02 – 0.06) | 0.40 |
| AInsulaL_SMGR | 0.24 (0.24) | 0.27 (0.21) | 0.02 (-0.02 – 0.07) | 0.32 |
| AInsulaR_RPFCL | 0.49 (0.24) | 0.51 (0.21) | 0.01 (-0.02 – 0.05) | 0.43 |
| AInsulaR_RPFCR | 0.34 (0.22) | 0.34 (0.23) | -0.003 (-0.05 – 0.04) | 0.91 |
| AInsulaR_SMGL | 0.20 (0.23) | 0.22 (0.23) | 0.02 (-0.02 – 0.07) | 0.35 |
| AInsulaR_SMGR | 0.42 (0.20) | 0.41 (0.18) | -0.002 (-0.04 – 0.04) | 0.91 |
| RPFCL_RPFCR | 0.20 (0.28) | 0.21 (0.27) | 0.01 (-0.04 – 0.06) | 0.73 |
| RPFCL_SMGL | 0.43 (0.23) | 0.45 (0.21) | 0.02 (-0.03 – 0.06) | 0.44 |
| RPFCL_SMGR | 0.33 (0.28) | 0.37 (0.22) | 0.04 (-0.01 – 0.10) | 0.12 |
| RPFCR_SMGL | 0.51 (0.20) | 0.54 (0.20) | 0.03 (-0.02 – 0.07) | 0.22 |
| RPFCR_SMGR | 0.38 (0.24) | 0.40 (0.23) | 0.02 (-0.03 – 0.07) | 0.39 |
| SMGL_SMGR | 0.30 (0.27) | 0.33 (0.22) | 0.03 (-0.02 – 0.07) | 0.29 |

Abbreviations: ACC – Anterior cingulate cortex; AInsula – Anterior insula; RPFC – Rostral prefrontal cortex; SMG – Supramarginal gyrus; nodes that end with L – left side; nodes that end with R – right side.

**eTable 4. Effect of doubling calligraphy practice on functional connectivity changes of salience network in younger and older age groups.**

| **Functional Connectivity** | **Younger age group** | | | | **Older age group** | | | |
| --- | --- | --- | --- | --- | --- | --- | --- | --- |
|  | **Intervention** | **Control** | **Intervention vs Control** | | **Intervention** | **Control** | **Intervention vs Control** | |
|  | **Mean change**  **(95% CI)** | **Mean change**  **(95% CI)** | **Mean difference**  **(95% CI)** | **p-value** | **Mean change**  **(95% CI)** | **Mean change**  **(95% CI)** | **Mean difference**  **(95% CI)** | **p-value** |
| ACC_AInsulaL | -0.02  (-0.12 – 0.08) | -0.06  (-0.14 – 0.03) | 0.03  (-0.09 – 0.16) | 0.60 | -0.07  (-0.16 – 0.03) | 0.04  (-0.07 – 0.14) | -0.10  (-0.24 – 0.03) | 0.14 |
| ACC_AInsulaR | 0.05  (-0.06 – 0.16) | 0.05  (-0.03 – 0.14) | -0.01  (-0.14 – 0.13) | 0.93 | -0.01  (-0.10 – 0.08) | -0.04  (-0.13 – 0.05) | 0.03  (-0.09 – 0.15) | 0.60 |
| ACC_RPFCL | 0.02  (-0.10 – 0.14) | 0.05  (-0.03 – 0.12) | -0.03  (-0.17 – 0.11) | 0.68 | 0.01  (-0.07 – 0.10) | 0.03  (-0.06 – 0.12) | -0.02  (-0.14 – 0.10) | 0.78 |
| ACC_RPFCR | 0.05  (-0.04 – 0.14) | 0.01  (-0.08 – 0.11) | 0.04  (-0.09 – 0.16) | 0.54 | -0.04  (-0.14 – 0.06) | 0.07  (-0.01 – 0.16) | -0.11  (-0.24 – 0.01) | 0.08 |
| ACC_SMGL | 0.01  (-0.08 – 0.11) | -0.03  (-0.11 – 0.04) | 0.05  (-0.07 – 0.16) | 0.40 | -0.02  (-0.12 – 0.07) | 0.02  (-0.10 – 0.14) | -0.04  (-0.19 – 0.11) | 0.57 |
| ACC_SMGR | -0.01  (-0.10 – 0.08) | -0.003  (-0.09 – 0.09) | -0.01  (-0.14 – 0.12) | 0.89 | 0.03  (-0.07 – 0.14) | -0.07  (-0.17 – 0.03) | 0.10  (-0.04 – 0.25) | 0.16 |
| AInsulaL_AInsulaR | -0.04  (-0.12 – 0.04) | -0.01  (-0.09 – 0.08) | -0.04  (-0.15 – 0.08) | 0.52 | 0.01  (-0.09 – 0.12) | 0.02  (-0.07 – 0.11) | -0.004  (-0.14 – 0.13) | 0.95 |
| AInsulaL_RPFCL | -0.02  (-0.11 – 0.08) | 0.08  (0.01 – 0.15) | -0.09  (-0.21 – 0.02) | 0.12 | 0.03  (-0.05 – 0.11) | 0.03  (-0.08 – 0.14) | -0.004  (-0.14 – 0.13) | 0.96 |
| AInsulaL_RPFCR | -0.01  (-0.10 – 0.08) | -0.01  (-0.09 – 0.06) | 0.004  (-0.11 – 0.12) | 0.95 | -0.03  (-0.12 – 0.06) | 0.03  (-0.06 – 0.13) | -0.06  (-0.19 – 0.06) | 0.32 |
| AInsulaL_SMGL | -0.0002  (-0.07 – 0.07) | 0.01  (-0.05 – 0.07) | -0.01  (-0.10 – 0.08) | 0.82 | 0.01  (-0.08 – 0.10) | 0.05  (-0.05 – 0.15) | -0.04  (-0.17 – 0.10) | 0.58 |
| AInsulaL_SMGR | 0.01  (-0.08 – 0.10) | -0.02  (-0.11 – 0.08) | 0.02  (-0.11 – 0.15) | 0.72 | 0.09  (-0.01 – 0.20) | 0.01  (-0.08 – 0.10) | 0.08  (-0.05 – 0.22) | 0.22 |
| AInsulaR_RPFCL | 0.05  (-0.03 – 0.13) | -0.05  (-0.11 – 0.02) | 0.10  (-0.004 – 0.20) | 0.06 | 0.04  (-0.02 – 0.11) | 0.01  (-0.06 – 0.08) | 0.03  (-0.06 – 0.12) | 0.54 |
| AInsulaR_RPFCR | 0.02  (-0.07 – 0.10) | -0.01  (-0.08 – 0.06) | 0.03  (-0.08 – 0.14) | 0.60 | -0.01  (-0.12 – 0.11) | -0.01  (-0.11 – 0.09) | 0.001  (-0.14 – 0.15) | 0.99 |
| AInsulaR_SMGL | 0.04  (-0.03 – 0.12) | -0.02  (-0.09 – 0.05) | 0.07  (-0.03 – 0.17) | 0.18 | -0.01  (-0.13 – 0.11) | 0.08  (-0.02 – 0.17) | -0.09  (-0.24 – 0.06) | 0.26 |
| AInsulaR_SMGR | -0.02  (-0.11 – 0.07) | -0.05  (-0.13 – 0.03) | 0.03  (-0.08 – 0.15) | 0.57 | 0.03  (-0.05 – 0.11) | 0.02  (-0.05 – 0.10) | 0.01  (-0.10 – 0.12) | 0.88 |
| RPFCL_RPFCR | 0.02  (-0.11 – 0.16) | -0.01  (-0.09 – 0.07) | 0.03  (-0.12 – 0.19) | 0.66 | -0.03  (-0.13 – 0.08) | 0.05  (-0.06 – 0.16) | -0.08  (-0.22 – 0.07) | 0.32 |
| RPFCL_SMGL | 0.03  (-0.05 – 0.11) | 0.004  (-0.05 – 0.06) | 0.03  (-0.07 – 0.12) | 0.58 | -0.03  (-0.13 – 0.06) | 0.07  (-0.04 – 0.19) | -0.11  (-0.26 – 0.05) | 0.17 |
| RPFCL_SMGR | 0.04  (-0.06 – 0.15) | -0.02  (-0.15 – 0.10) | 0.07  (-0.10 – 0.23) | 0.42 | 0.08  (-0.03 – 0.18) | 0.07  (-0.02 – 0.16) | 0.002  (-0.13 – 0.13) | 0.98 |
| RPFCR_SMGL | 0.03  (-0.05 – 0.11) | -0.001  (-0.09 – 0.08) | 0.03  (-0.08 – 0.14) | 0.56 | -0.01  (-0.09 – 0.06) | 0.10  (-0.02 – 0.22) | -0.11  (-0.25 – 0.03) | 0.12 |
| RPFCR_SMGR | 0.02  (-0.07 – 0.11) | -0.04  (-0.10 – 0.03) | 0.06  (-0.05 – 0.17) | 0.26 | 0.14  (0.003 – 0.28) | -0.05  (-0.12 – 0.03) | 0.19  (0.03 – 0.35) | **0.02** |
| SMGL_SMGR | 0.01  (-0.06 – 0.08) | -0.06  (-0.17 – 0.05) | 0.06  (-0.06 – 0.19) | 0.32 | 0.12  (0.02 – 0.23) | 0.03  (-0.06 – 0.12) | 0.09  (-0.04 – 0.23) | 0.17 |

Abbreviations: ACC – Anterior cingulate cortex; AInsula – Anterior insula; RPFC – Rostral prefrontal cortex; SMG – Supramarginal gyrus; nodes that end with L – left side; nodes that end with R – right side

**eTable 5. Effect of doubling calligraphy practice on functional connectivity changes of salience network in female and male groups.**

| **Functional Connectivity** | **Female** | | | | **Male** | | | |
| --- | --- | --- | --- | --- | --- | --- | --- | --- |
|  | **Intervention** | **Control** | **Intervention vs Control** | | **Intervention** | **Control** | **Intervention vs Control** | |
|  | **Mean change**  **(95% CI)** | **Mean change**  **(95% CI)** | **Mean difference**  **(95% CI)** | **p-value** | **Mean change**  **(95% CI)** | **Mean change**  **(95% CI)** | **Mean difference**  **(95% CI)** | **p-value** |
| ACC_AInsulaL | -0.01  (-0.08 – 0.06) | 0.01  (-0.06 – 0.09) | -0.03  (-0.12 – 0.07) | 0.61 | -0.13  (-0.31 – 0.04) | -0.08  (-0.24 – 0.08) | -0.05  (-0.28 – 0.17) | 0.63 |
| ACC_AInsulaR | 0.04  (-0.03 – 0.12) | 0.02  (-0.05 – 0.09) | 0.02  (-0.08 – 0.12) | 0.69 | -0.05  (-0.20 – 0.11) | -0.04  (-0.16 – 0.08) | -0.01  (-0.21 – 0.20) | 0.96 |
| ACC_RPFCL | 0.04  (-0.02 – 0.11) | 0.06  (­-0.01 – 0.12) | -0.01  (-0.10 – 0.07) | 0.75 | -0.05  (-0.23 – 0.13) | -0.01  (-0.15 – 0.13) | -0.04  (-0.28 – 0.20) | 0.73 |
| ACC_RPFCR | 0.03  (-0.04 – 0.11) | 0.06  (-0.01 – 0.14) | -0.03  (-0.14 – 0.07) | 0.56 | -0.07  (-0.20 – 0.06) | -0.02  (-0.13 – 0.09) | -0.05  (-0.21 – 0.11) | 0.54 |
| ACC_SMGL | 0.04  (-0.02 – 0.10) | -0.02  (-0.10 – 0.07) | 0.06  (-0.05 – 0.16) | 0.29 | -0.12  (-0.29 – 0.05) | 0.02  (-0.09 – 0.14) | -0.14  (-0.34 – 0.06) | 0.16 |
| ACC_SMGR | 0.08  (0.01 – 0.15) | -0.01  (-0.08 – 0.06) | 0.09  (-0.004 – 0.19) | 0.06 | -0.18  (-0.33 – -0.03) | -0.12  (-0.28 – 0.05) | -0.07  (-0.29 – 0.16) | 0.56 |
| AInsulaL_AInsulaR | 0.004  (-0.05 – 0.06) | 0.03  (-0.04 – 0.09) | -0.02  (-0.11 – 0.06) | 0.62 | -0.07  (-0.27 – 0.13) | -0.05  (-0.19 – 0.09) | -0.01  (-0.25 – 0.22) | 0.90 |
| AInsulaL_RPFCL | 0.04  (-0.02 – 0.10) | 0.06  (-0.02 – 0.14) | -0.02  (-0.11 – 0.08) | 0.75 | -0.09  (-0.25 – 0.07) | 0.05  (-0.08 – 0.18) | -0.14  (-0.34 – 0.06) | 0.18 |
| AInsulaL_RPFCR | -0.01  (-0.08 – 0.06) | 0.03  (-0.05 – 0.10) | -0.04  (-0.14 – 0.06) | 0.45 | -0.05  (-0.19 – 0.09) | -0.05  (-0.15 – 0.05) | -0.003  (-0.17 – 0.16) | 0.97 |
| AInsulaL_SMGL | 0.01  (-0.05 – 0.07) | 0.02  (-0.05 – 0.09) | -0.01  (-0.10 – 0.08) | 0.79 | -0.01  (-0.13 – 0.12) | 0.05  (-0.05 – 0.15) | -0.06  (-0.21 – 0.10) | 0.47 |
| AInsulaL_SMGR | 0.07  (-0.01 – 0.15) | 0.01  (-0.06 – 0.08) | 0.05  (-0.05 – 0.16) | 0.31 | 0.01  (-0.14 – 0.16) | -0.05  (-0.21 – 0.12) | 0.06  (-0.15 – 0.27) | 0.58 |
| AInsulaR_RPFCL | 0.06  (0.004 – 0.12) | -0.03  (-0.09 – 0.03) | 0.09  (0.01 – 0.18) | **0.03** | -0.01  (-0.11 – 0.10) | 0.01  (-0.06 – 0.09) | -0.02  (-0.14 – 0.10) | 0.75 |
| AInsulaR_RPFCR | 0.02  (-0.06 – 0.10) | -0.01  (-0.08 – 0.05) | 0.04  (-0.06 – 0.14) | 0.47 | -0.05  (-0.19 – 0.10) | 0.004  (-0.13 – 0.13) | -0.05  (-0.24 – 0.14) | 0.61 |
| AInsulaR_SMGL | 0.03  (-0.04 – 0.10) | 0.03  (-0.04 – 0.10) | -0.001  (-0.10 – 0.10) | 0.99 | -0.01  (-0.17 – 0.14) | 0.02  (-0.10 – 0.13) | -0.03  (-0.24 – 0.18) | 0.75 |
| AInsulaR_SMGR | 0.02  (-0.04 – 0.09) | -0.03  (-0.10 – 0.03) | 0.06  (-0.03 – 0.15) | 0.22 | -0.03  (-0.17 – 0.11) | 0.05  (-0.06 – 0.16) | -0.08  (-0.25 – 0.09) | 0.33 |
| RPFCL_RPFCR | 0.04  (-0.06 – 0.13) | 0.03  (-0.05 – 0.11) | 0.01  (-0.12 – 0.13) | 0.93 | -0.10  (-0.26 – 0.06) | -0.01  (-0.14 – 0.12) | -0.09  (-0.29 – 0.11) | 0.37 |
| RPFCL_SMGL | -0.02  (-0.09 – 0.05) | 0.06  (-0.02 – 0.15) | -0.08  (-0.19 – 0.02) | 0.13 | 0.04  (-0.09 – 0.16) | -0.04  (-0.17 – 0.09) | 0.08  (-0.09 – 0.25) | 0.36 |
| RPFCL_SMGR | 0.08  (0.001 – 0.17) | 0.03  (-0.05 – 0.11) | 0.05  (-0.06 – 0.17) | 0.37 | -0.01  (-0.16 – 0.14) | 0.003  (-0.18 – 0.18) | -0.02  (-0.24 – 0.21) | 0.89 |
| RPFCR_SMGL | 0.02  (-0.05 – 0.08) | 0.06  (-0.03 – 0.16) | -0.05  (-0.16 – 0.06) | 0.39 | -0.01  (-0.13 – 0.10) | -0.003  (-0.11 – 0.11) | -0.01  (-0.16 – 0.14) | 0.90 |
| RPFCR_SMGR | 0.09  (-0.01 – 0.20) | -0.04  (-0.10 – 0.01) | 0.13  (0.02 – 0.25) | **0.02** | 0.06  (-0.09 – 0.20) | -0.04  (-0.15 – 0.07) | 0.10  (-0.08 – 0.27) | 0.27 |
| SMGL_SMGR | 0.06  (-0.01 – 0.14) | 0.01  (-0.07 – 0.09) | 0.05  (-0.06 – 0.16) | 0.34 | 0.07  (-0.05 – 0.20) | -0.08  (-0.25 – 0.08) | 0.16  (-0.04 – 0.35) | 0.11 |

Abbreviations: ACC – Anterior cingulate cortex; AInsula – Anterior insula; RPFC – Rostral prefrontal cortex; SMG – Supramarginal gyrus; nodes that end with L – left side; nodes that end with R – right side.

**eTable 6. Effect of doubling calligraphy practice on functional connectivity changes of salience network in groups with fewer and more years of education.**

| **Functional Connectivity** | **Fewer years of education** | | | | **More years of education** | | | |
| --- | --- | --- | --- | --- | --- | --- | --- | --- |
|  | **Intervention** | **Control** | **Intervention vs Control** | | **Intervention** | **Control** | **Intervention vs Control** | |
|  | **Mean change**  **(95% CI)** | **Mean change**  **(95% CI)** | **Mean difference**  **(95% CI)** | **p-value** | **Mean change**  **(95% CI)** | **Mean change**  **(95% CI)** | **Mean difference**  **(95% CI)** | **p-value** |
| ACC_AInsulaL | -0.06  (-0.14 – 0.03) | -0.04  (-0.15 – 0.08) | -0.02  (-0.16 – 0.12) | 0.78 | -0.04  (-0.15 – 0.07) | 0.01  (-0.05 – 0.08) | -0.05  (-0.18 – 0.08) | 0.43 |
| ACC_AInsulaR | -0.002  (-0.10 – 0.09) | -0.05  (-0.14 – 0.04) | 0.05  (-0.08 – 0.18) | 0.47 | 0.04  (-0.07 – 0.15) | 0.06  (-0.02 – 0.14) | -0.02  (-0.15 – 0.11) | 0.74 |
| ACC_RPFCL | -0.03  (-0.12 – 0.07) | 0.05  (-0.03 – 0.12) | -0.07  (-0.19 – 0.05) | 0.23 | 0.06  (-0.04 – 0.16) | 0.03  (-0.06 – 0.12) | 0.03  (-0.11 – 0.16) | 0.67 |
| ACC_RPFCR | 0.01  (-0.09 – 0.10) | 0.03  (-0.06 – 0.13) | -0.02  (-0.16 – 0.11) | 0.72 | 0.002  (-0.09 – 0.09) | 0.05  (-0.04 – 0.14) | -0.05  (-0.17 – 0.07) | 0.41 |
| ACC_SMGL | -0.02  (-0.11 – 0.08) | -0.02  (-0.13 – 0.08) | 0.01  (-0.13 – 0.15) | 0.90 | 0.01  (-0.09 – 0.10) | 0.01  (-0.08 – 0.10) | -0.003  (-0.13 – 0.13) | 0.97 |
| ACC_SMGR | 0.01  (-0.09 – 0.11) | -0.15  (-0.25 – -0.04) | 0.16  (0.01 – 0.30) | **0.03** | 0.01  (-0.09 – 0.11) | 0.07  (0.01 – 0.13) | -0.06  (-0.18 – 0.06) | 0.30 |
| AInsulaL_AInsulaR | -0.03  (-0.10 – 0.05) | 0.04  (-0.05 – 0.13) | -0.06  (-0.18 – 0.05) | 0.26 | -0.002  (-0.11 – 0.11) | -0.03  (-0.11 – 0.06) | 0.02  (-0.11 – 0.16) | 0.73 |
| AInsulaL_RPFCL | 0.05  (-0.03 – 0.13) | 0.11  (0.004 – 0.21) | -0.06  (-0.19 – 0.07) | 0.37 | -0.03  (-0.13 – 0.06) | 0.004  (-0.08 – 0.09) | -0.04  (-0.16 – 0.08) | 0.53 |
| AInsulaL_RPFCR | -0.03  (-0.13 – 0.06) | 0.09  (-0.01 – 0.18) | -0.12  (-0.25 – 0.02) | 0.08 | -0.01  (-0.09 – 0.07) | -0.07  (-0.13 – -0.01) | 0.06  (-0.04 – 0.16) | 0.25 |
| AInsulaL_SMGL | 0.02  (-0.06 – 0.09) | 0.02  (-0.07 – 0.12) | -0.01  (-0.12 – 0.11) | 0.90 | -0.01  (-0.09 – 0.08) | 0.03  (-0.04 – 0.10) | -0.04  (-0.15 – 0.07) | 0.46 |
| AInsulaL_SMGR | 0.07  (-0.03 – 0.17) | 0.06  (-0.03 – 0.15) | 0.01  (-0.12 – 0.14) | 0.89 | 0.03  (-0.07 – 0.13) | -0.07  (-0.16 – 0.02) | 0.10  (-0.03 – 0.23) | 0.14 |
| AInsulaR_RPFCL | 0.07  (0.01 – 0.14) | -0.01  (-0.09 – 0.06) | 0.09  (-0.01 – 0.19) | 0.09 | 0.02  (-0.06 – 0.09) | -0.02  (-0.08 – 0.04) | 0.04  (-0.05 – 0.14) | 0.39 |
| AInsulaR_RPFCR | -0.05  (-0.16 – 0.06) | -0.0002  (-0.09 – 0.09) | -0.05  (-0.19 – 0.09) | 0.51 | 0.06  (-0.03 – 0.14) | -0.02  (-0.10 – 0.06) | 0.08  (-0.04 – 0.19) | 0.19 |
| AInsulaR_SMGL | 0.03  (-0.05 – 0.12) | 0.03  (-0.04 – 0.11) | 0.001  (-0.11 – 0.11) | 0.99 | 0.001  (-0.11 – 0.12) | 0.02  (-0.08 – 0.12) | -0.02  (-0.17 – 0.13) | 0.79 |
| AInsulaR_SMGR | 0.01  (-0.06 – 0.09) | 0.01  (-0.09 – 0.10) | 0.01  (-0.11 – 0.12) | 0.89 | 0.003  (-0.09 – 0.10) | -0.03  (-0.10 – 0.04) | 0.03  (-0.08 – 0.15) | 0.56 |
| RPFCL_RPFCR | -0.02  (-0.16 – 0.12) | 0.01  (-0.09 – 0.10) | -0.03  (-0.19 – 0.13) | 0.73 | 0.02  (-0.09 – 0.12) | 0.03  (-0.07 – 0.13) | -0.01  (-0.15 – 0.13) | 0.85 |
| RPFCL_SMGL | -0.001  (-0.09 – 0.09) | 0.02  (-0.09 – 0.13) | -0.02  (-0.16 – 0.12) | 0.77 | -0.003  (-0.09 – 0.08) | 0.06  (-0.03 – 0.15) | -0.06  (-0.18 – 0.06) | 0.32 |
| RPFCL_SMGR | 0.06  (-0.03 – 0.16) | 0.03  (-0.09 – 0.15) | 0.04  (-0.12 – 0.19) | 0.63 | 0.05  (-0.06 – 0.16) | 0.02  (-0.07 – 0.12) | 0.03  (-0.11 – 0.17) | 0.67 |
| RPFCR_SMGL | -0.02  (-0.10 – 0.06) | 0.06  (-0.06 – 0.19) | -0.09  (-0.24 – 0.06) | 0.25 | 0.04  (-0.03 – 0.11) | 0.03 (-0.04 – 0.10) | 0.01  (-0.09 – 0.11) | 0.84 |
| RPFCR_SMGR | 0.04  (-0.08 – 0.16) | -0.03  (-0.10 – 0.04) | 0.07  (-0.07 – 0.21) | 0.31 | 0.13  (0.01 – 0.25) | -0.05 (-0.12 – 0.01) | 0.18  (0.05 – 0.31) | **0.01** |
| SMGL_SMGR | 0.02  (-0.07 – 0.10) | -0.01  (-0.13 – 0.10) | 0.03  (-0.11 – 0.17) | 0.68 | 0.11  (0.02 – 0.21) | -0.02  (-0.11 – 0.08) | 0.13  (0.001 – 0.26) | **0.049** |

Abbreviations: ACC – Anterior cingulate cortex; AInsula – Anterior insula; RPFC – Rostral prefrontal cortex; SMG – Supramarginal gyrus; nodes that end with L – left side; nodes that end with R – right side.

**eTable 7. Effect of doubling calligraphy practice on functional connectivity changes of salience network in groups with fewer and more of prior practice.**

| **Functional Connectivity** | **Fewer years of prior practice** | | | | **More years of prior practice** | | | |
| --- | --- | --- | --- | --- | --- | --- | --- | --- |
|  | **Intervention** | **Control** | **Intervention vs Control** | | **Intervention** | **Control** | **Intervention vs Control** | |
|  | **Mean change**  **(95% CI)** | **Mean change**  **(95% CI)** | **Mean difference**  **(95% CI)** | **p-value** | **Mean change**  **(95% CI)** | **Mean change**  **(95% CI)** | **Mean difference**  **(95% CI)** | **p-value** |
| ACC_AInsulaL | -0.09  (-0.18 – -0.004) | -0.03  (-0.13 – 0.08) | -0.07  (-0.20 – 0.07) | 0.31 | 0.001  (-0.10 – 0.10) | 0.003  (-0.09 – 0.09) | -0.003  (-0.14 – 0.13) | 0.97 |
| ACC_AInsulaR | -0.03  (-0.14 – 0.08) | 0.02  (-0.07 – 0.10) | -0.05  (-0.18 – 0.09) | 0.48 | 0.07  (-0.02 – 0.16) | -0.004  (-0.10 – 0.09) | 0.07  (-0.05 – 0.20) | 0.25 |
| ACC_RPFCL | -0.05  (-0.17 – 0.07) | -0.01  (-0.09 – 0.07) | -0.04  (-0.18 – 0.10) | 0.54 | 0.08  (0.01 – 0.16) | 0.09  (0.004 – 0.17) | -0.002  (-0.11 – 0.11) | 0.96 |
| ACC_RPFCR | -0.0003  (-0.10 – 0.10) | 0.06  (-0.05 – 0.16) | -0.06  (-0.20 – 0.09) | 0.43 | 0.01  (-0.07 – 0.09) | 0.03  (-0.05 – 0.10) | -0.02  (-0.13 – 0.09) | 0.75 |
| ACC_SMGL | 0.01  (-0.09 – 0.11) | 0.001  (-0.11 – 0.11) | 0.01  (-0.14 – 0.15) | 0.94 | -0.01  (-0.10 – 0.07) | -0.01  (-0.10 – 0.07) | -0.0001  (-0.12 – 0.12) | 0.99 |
| ACC_SMGR | 0.01  (-0.08 – 0.09) | -0.05  (-0.16 – 0.05) | 0.06  (-0.07 – 0.19) | 0.36 | 0.02  (-0.09 – 0.12) | -0.02  (-0.11 – 0.07) | 0.04  (-0.11 – 0.18) | 0.62 |
| AInsulaL_AInsulaR | -0.08  (-0.16 – 0.01) | -0.03  (-0.12 – 0.06) | -0.04  (-0.16 – 0.07) | 0.46 | 0.05  (-0.05 – 0.14) | 0.04  (-0.04 – 0.13) | 0.002  (-0.12 – 0.13) | 0.98 |
| AInsulaL_RPFCL | 0.01  (-0.08 – 0.11) | 0.03  (-0.07 – 0.13) | -0.02  (-0.15 – 0.12) | 0.82 | 0.001  (-0.08 – 0.08) | 0.08  (-0.01 – 0.17) | -0.08  (-0.20 – 0.03) | 0.17 |
| AInsulaL_RPFCR | -0.05  (-0.13 – 0.04) | -0.003  (-0.10 – 0.09) | -0.04  (-0.17 – 0.08) | 0.50 | 0.004  (-0.08 – 0.09) | 0.02  (-0.06 – 0.10) | -0.02  (-0.13 – 0.10) | 0.78 |
| AInsulaL_SMGL | 0.02  (-0.04 – 0.09) | 0.002  (-0.09 – 0.09) | 0.02  (-0.09 – 0.13) | 0.71 | -0.01  (-0.10 – 0.08) | 0.06  (-0.02 – 0.13) | -0.07 (-0.18 – 0.05) | 0.23 |
| AInsulaL_SMGR | 0.07  (-0.04 – 0.18) | -0.04  (-0.14 – 0.06) | 0.11  (-0.03 – 0.26) | 0.12 | 0.03  (-0.06 – 0.12) | 0.04  (-0.05 – 0.12) | -0.01  (-0.13 – 0.11) | 0.91 |
| AInsulaR_RPFCL | 0.03  (-0.04 – 0.10) | -0.01  (-0.07 – 0.06) | 0.04  (-0.06 – 0.13) | 0.46 | 0.06  (-0.01 – 0.14) | -0.03  (-0.10 – 0.04) | 0.09  (-0.01 – 0.19) | 0.07 |
| AInsulaR_RPFCR | -0.03  (-0.13 – 0.07) | -0.003  (-0.10 – 0.09) | -0.03  (-0.16 – 0.11) | 0.71 | 0.04  (-0.06 – 0.13) | -0.02  (-0.09 – 0.05) | 0.06  (-0.06 – 0.17) | 0.35 |
| AInsulaR_SMGL | -0.02  (-0.10 – 0.07) | 0.02  (-0.07 – 0.11) | -0.04  (-0.16 – 0.08) | 0.54 | 0.05  (-0.06 – 0.16) | 0.03  (-0.05 – 0.12) | 0.02  (-0.12 – 0.16) | 0.79 |
| AInsulaR_SMGR | -0.002  (-0.10 – 0.10) | -0.02  (-0.10 – 0.07) | 0.02  (-0.11 – 0.14) | 0.81 | 0.02  (-0.06 – 0.09) | -0.01  (-0.08 – 0.07) | 0.03  (-0.08 – 0.13) | 0.60 |
| RPFCL_RPFCR | -0.03  (-0.18 – 0.12) | 0.02  (-0.07 – 0.10) | -0.05  (-0.21 – 0.12) | 0.57 | 0.03  (-0.06 – 0.11) | 0.02  (-0.09 – 0.13) | 0.01  (-0.13 – 0.14) | 0.93 |
| RPFCL_SMGL | 0.01  (-0.08 – 0.10) | 0.02  (-0.07 – 0.10) | -0.01  (-0.13 – 0.12) | 0.93 | -0.01  (-0.10 – 0.07) | 0.06  (-0.05 – 0.17) | -0.08  (-0.21 – 0.06) | 0.27 |
| RPFCL_SMGR | 0.12  (-0.003 – 0.24) | 0.01  (-0.12 – 0.14) | 0.11  (-0.06 – 0.28) | 0.21 | -0.001  (-0.08 – 0.08) | 0.04  (-0.04 – 0.13) | -0.04  (-0.16 – 0.07) | 0.45 |
| RPFCR_SMGL | -0.03  (-0.12 – 0.06) | 0.06  (-0.02 – 0.14) | -0.09  (-0.20 – 0.03) | 0.15 | 0.05  (-0.01 – 0.11) | 0.04  (-0.08 – 0.16) | 0.01  (-0.13 – 0.14) | 0.89 |
| RPFCR_SMGR | 0.11  (-0.03 – 0.24) | -0.04  (-0.11 – 0.03) | 0.15  (-0.001 – 0.30) | 0.05 | 0.06  (-0.04 – 0.16) | -0.04  (-0.11 – 0.03) | 0.10  (-0.02 – 0.22) | 0.10 |
| SMGL_SMGR | 0.06  (-0.04 – 0.17) | -0.06  (-0.17 – 0.05) | 0.13  (-0.02 – 0.27) | 0.09 | 0.07  (-0.02 – 0.15) | 0.03  (-0.06 – 0.13) | 0.03  (-0.09 – 0.15) | 0.60 |

Abbreviations: ACC – Anterior cingulate cortex; AInsula – Anterior insula; RPFC – Rostral prefrontal cortex; SMG – Supramarginal gyrus; nodes that end with L – left side; nodes that end with R – right side.

**eTable 8. Effect of doubling calligraphy practice on functional connectivity changes of salience network in groups of lesser and greater brain atrophy.**

| **Functional Connectivity** | **Lesser brain atrophy** | | | | **Greater brain atrophy** | | | |
| --- | --- | --- | --- | --- | --- | --- | --- | --- |
|  | **Intervention** | **Control** | **Intervention vs Control** | | **Intervention** | **Control** | **Intervention vs Control** | |
|  | **Mean change**  **(95% CI)** | **Mean change**  **(95% CI)** | **Mean difference**  **(95% CI)** | **p-value** | **Mean change**  **(95% CI)** | **Mean change**  **(95% CI)** | **Mean difference**  **(95% CI)** | **p-value** |
| ACC_AInsulaL | -0.06  (-0.15 – 0.03) | 0.05  (-0.05 – 0.16) | -0.11  (-0.24 – 0.02) | 0.11 | -0.03  (-0.14 – 0.07) | -0.08  (-0.16 – 0.01) | 0.04  (-0.09 – 0.17) | 0.54 |
| ACC_AInsulaR | 0.04  (-0.04 – 0.13) | 0.06  (-0.02 – 0.14) | -0.01  (-0.13 – 0.10) | 0.82 | -0.01  (-0.12 – 0.11) | -0.04  (-0.14 – 0.05) | 0.04  (-0.10 – 0.18) | 0.59 |
| ACC_RPFCL | 0.05  (-0.04 – 0.14) | 0.06  (-0.03 – 0.15) | -0.01  (-0.14 – 0.11) | 0.84 | -0.01  (-0.12 – 0.10) | 0.02  (-0.06 – 0.10) | -0.03  (-0.17 – 0.10) | 0.63 |
| ACC_RPFCR | -0.002  (-0.10 – 0.10) | 0.06  (-0.05 – 0.16) | -0.06  (-0.20 – 0.08) | 0.42 | 0.01  (-0.07 – 0.10) | 0.03  (-0.05 – 0.11) | -0.02  (-0.13 – 0.10) | 0.76 |
| ACC_SMGL | -0.01  (-0.09 – 0.08) | -0.01  (-0.11 – 0.09) | 0.005  (-0.12 – 0.13) | 0.94 | -0.001  (-0.10 – 0.10) | -0.002 (-0.10 – 0.10) | 0.001  (-0.14 – 0.14) | 0.99 |
| ACC_SMGR | 0.01  (-0.09 – 0.12) | -0.01  (-0.10 – 0.08) | 0.02  (-0.11 – 0.16) | 0.75 | 0.01  (-0.10 – 0.11) | -0.07  (-0.17 – 0.03) | 0.07  (-0.07 – 0.22) | 0.29 |
| AInsulaL_AInsulaR | 0.01  (-0.10 – 0.11) | 0.06  (-0.02 – 0.13) | -0.05  (-0.17 – 0.08) | 0.44 | -0.04  (-0.12 – 0.04) | -0.05  (-0.14 – 0.05) | 0.01  (-0.11 – 0.13) | 0.91 |
| AInsulaL_RPFCL | 0.01  (-0.10 – 0.11) | 0.08  (-0.03 – 0.19) | -0.08  (-0.22 – 0.07) | 0.30 | 0.01  (-0.06 – 0.08) | 0.03  (-0.05 – 0.10) | -0.02  (-0.12 – 0.08) | 0.72 |
| AInsulaL_RPFCR | -0.01  (-0.11 – 0.08) | 0.03  (-0.03 – 0.10) | -0.04  (-0.16 – 0.07) | 0.43 | -0.03  (-0.11 – 0.05) | -0.01  (-0.12 – 0.09) | -0.01  (-0.14 – 0.11) | 0.82 |
| AInsulaL_SMGL | -0.004  (-0.07 – 0.07) | 0.05  (-0.02 – 0.13) | -0.06  (-0.16 – 0.04) | 0.26 | 0.01  (-0.07 – 0.10) | 0.003  (-0.09 – 0.09) | 0.01  (-0.11 – 0.13) | 0.86 |
| AInsulaL_SMGR | 0.01  (-0.09 – 0.10) | 0.02  (-0.06 – 0.10) | -0.01  (-0.13 – 0.11) | 0.87 | 0.09  (-0.01 – 0.19) | -0.02  (-0.13 – 0.08) | 0.12  (-0.02 – 0.26) | 0.10 |
| AInsulaR_RPFCL | 0.06  (-0.02 – 0.15) | 0.02  (-0.03 – 0.08) | 0.04  (-0.06 – 0.14) | 0.45 | 0.03  (-0.03 – 0.08) | -0.06  (-0.13 – 0.01) | 0.09  (-0.002 – 0.18) | 0.06 |
| AInsulaR_RPFCR | 0.03  (-0.09 – 0.14) | -0.03  (-0.11 – 0.05) | 0.06  (-0.08 – 0.20) | 0.41 | -0.02  (-0.10 – 0.06) | 0.01  (-0.08 – 0.10) | -0.03  (-0.15 – 0.09) | 0.63 |
| AInsulaR_SMGL | 0.08  (-0.01 – 0.17) | 0.07  (-0.02 – 0.15) | 0.01  (-0.11 – 0.14) | 0.83 | -0.05  (-0.15 – 0.06) | -0.01  (-0.09 – 0.07) | -0.03  (-0.16 – 0.10) | 0.61 |
| AInsulaR_SMGR | -0.01  (-0.10 – 0.08) | -0.05  (-0.15 – 0.04) | 0.04  (-0.08 – 0.17) | 0.49 | 0.03  (-0.06 – 0.11) | 0.03  (-0.03 – 0.09) | -0.002  (-0.10 – 0.10) | 0.96 |
| RPFCL_RPFCR | 0.05  (-0.09 – 0.18) | 0.02  (-0.08 – 0.13) | 0.02  (-0.14 – 0.19) | 0.78 | -0.05  (-0.15 – 0.06) | 0.02  (-0.07 – 0.11) | -0.06  (-0.20 – 0.07) | 0.35 |
| RPFCL_SMGL | 0.04  (-0.04 – 0.13) | 0.03  (-0.07 – 0.14) | 0.01  (-0.13 – 0.14) | 0.90 | -0.05  (-0.13 – 0.04) | 0.04  (-0.05 – 0.13) | -0.09  (-0.21 – 0.03) | 0.14 |
| RPFCL_SMGR | 0.03  (-0.08 – 0.14) | 0.05  (-0.05 – 0.15) | -0.02  (-0.16 – 0.12) | 0.80 | 0.09  (-0.01 – 0.19) | 0.002  (­-0.12 – 0.12) | 0.09  (-0.07 – 0.24) | 0.26 |
| RPFCR_SMGL | 0.03  (-0.05 – 0.10) | 0.10  (0.01 – 0.19) | -0.07  (-0.19 – 0.04) | 0.20 | -0.01  (-0.09 – 0.07) | -0.01  (-0.12 – 0.10) | -0.002  (-0.14 – 0.13) | 0.97 |
| RPFCR_SMGR | 0.06  (-0.05 – 0.16) | -0.03  (-0.10 – 0.04) | 0.09  (-0.03 – 0.21) | 0.15 | 0.11  (-0.02 – 0.24) | -0.05  (-0.12 – 0.02) | 0.16  (0.01 – 0.31) | **0.03** |
| SMGL_SMGR | 0.06  (-0.03 – 0.15) | 0.04  (-0.06 – 0.14) | 0.02  (-0.11 – 0.16) | 0.76 | 0.07  (-0.02 – 0.16) | -0.07  (-0.17 – 0.03) | 0.14  (0.01 – 0.27) | **0.04** |

Abbreviations: ACC – Anterior cingulate cortex; AInsula – Anterior insula; RPFC – Rostral prefrontal cortex; SMG – Supramarginal gyrus; nodes that end with L – left side; nodes that end with R – right side.
